# Supplementary material for: Downregulation of the tyrosine degradation pathway extends Drosophila lifespan
Source: eLife. 2020 Dec 15;9:e58053. doi: 10.7554/eLife.58053 (PMC7744100; doi:10.7554/eLife.58053)
Supplement: Transparent reporting form [file elife-58053-transrepform.docx]

***eLife’s* transparent reporting form**

We encourage authors to provide detailed information *within their submission* to facilitate the interpretation and replication of experiments. Authors can upload supporting documentation to indicate the use of appropriate reporting guidelines for health-related research (see [EQUATOR Network](http://www.equator-network.org/%20)), life science research (see the [BioSharing Information Resource](https://biosharing.org/)), or the [ARRIVE guidelines](http://www.plosbiology.org/article/info:doi/10.1371/journal.pbio.1000412) for reporting work involving animal research. Where applicable, authors should refer to any relevant reporting standards documents in this form.

If you have any questions, please consult our Journal Policies and/or contact us: [editorial@elifesciences.org](mailto:editorial@elifesciences.org).

**Sample-size estimation**

- You should state whether an appropriate sample size was computed when the study was being designed
- You should state the statistical method of sample size computation and any required assumptions
- If no explicit power analysis was used, you should describe how you decided what sample (replicate) size (number) to use

Please outline where this information can be found within the submission (e.g., sections or figure legends), or explain why this information doesn’t apply to your submission:

We did not use any sample size computation nor power analysis. We decided to have at least 3-5 independent biological replicates per condition based on our previous experiments with qRT-PCR (except aging experiment, where we used 8 biological replecates, see below), metabolomics and measurements of neuromediators. For lifespan experiments, we used the number of flies that far exceeds the number of flies to detect 10% in lifespan using log-rank test.

**Replicates**

- You should report how often each experiment was performed
- You should include a definition of biological versus technical replication
- The data obtained should be provided and sufficient information should be provided to indicate the number of independent biological and/or technical replicates
- If you encountered any outliers, you should describe how these were handled
- Criteria for exclusion/inclusion of data should be clearly stated
- High-throughput sequence data should be uploaded before submission, with a private link for reviewers provided (these are available from both GEO and ArrayExpress)

Please outline where this information can be found within the submission (e.g., sections or figure legends), or explain why this information doesn’t apply to your submission:

All qRT-PCR experiments were performed with 3-5 biological replicates per condition, except Figure 1D, where we used 8 biological replicates per condition (due to high noise in aged flies). Outliers were excluded in the case of problems with sample preparation (when mRNA levels of a housekeeping gene are dramatically lower than of other samples in the same group).

All metabolomics experiments were performed with 4-5 biological replicates per condition. The sample was excluded from the analysis if during sample preparation it was crashed/spilled during the grinding process.

All neurotransmitter experiments were performed with 3 biological replicates per condition.

All lifespan experiments were performed with 150 – 250 flies per condition and lifespan data were pooled together.

All stress assays were performed with 50-60 flies per condition and lifespan data were pooled together.

**Statistical reporting**

- Statistical analysis methods should be described and justified
- Raw data should be presented in figures whenever informative to do so (typically when N per group is less than 10)
- For each experiment, you should identify the statistical tests used, exact values of N, definitions of center, methods of multiple test correction, and dispersion and precision measures (e.g., mean, median, SD, SEM, confidence intervals; and, for the major substantive results, a measure of effect size (e.g., Pearson's r, Cohen's d)
- Report exact p-values wherever possible alongside the summary statistics and 95% confidence intervals. These should be reported for all key questions and not only when the p-value is less than 0.05.

Please outline where this information can be found within the submission (e.g., sections or figure legends), or explain why this information doesn’t apply to your submission:

All the statistics for the replicates mentioned above were performed using JMP SAS Pro 12 and Excel. The “Statistics” section in Methods describes the statistical methods used and the detailed information with regards to the t-test and the error bars (SEM or SD) can be found in the figure legends wherever necessary. Statistical analysis of metabolomics data was performed in Metaboanalyst.ca and described in Methods. All lifespan counts can be found in the Supplemental table.

(For large datasets, or papers with a very large number of statistical tests, you may upload a single table file with tests, Ns, etc., with reference to sections in the manuscript.)

**Group allocation**

- Indicate how samples were allocated into experimental groups (in the case of clinical studies, please specify allocation to treatment method); if randomization was used, please also state if restricted randomization was applied
- Indicate if masking was used during group allocation, data collection and/or data analysis

Please outline where this information can be found within the submission (e.g., sections or figure legends), or explain why this information doesn’t apply to your submission:

N.A.

**Additional data files (“source data”)**

- We encourage you to upload relevant additional data files, such as numerical data that are represented as a graph in a figure, or as a summary table
- Where provided, these should be in the most useful format, and they can be uploaded as “Source data” files linked to a main figure or table
- Include model definition files including the full list of parameters used
- Include code used for data analysis (e.g., R, MatLab)
- Avoid stating that data files are “available upon request”

Please indicate the figures or tables for which source data files have been provided:

N.A.
